# Tris(2,3-dibromopropyl)Isocyanurate has an Effect on Inflammation Markers in Mouse Primary Astrocytes *In vitro*

**DOI:** 10.1007/s10753-023-01837-y

**Published:** 2023-05-25

**Authors:** Konrad A. Szychowski, Bartosz Skóra

**Affiliations:** Department of Biotechnology and Cell Biology, Medical College, University of Information Technology and Management in Rzeszow, Sucharskiego 2, 35-225, Rzeszow, Poland

**Keywords:** TBC, inflammation, astrocyte, apoptosis, toxicity, ROS.

## Abstract

Tris(2,3-dibromopropyl)isocyanurate (TBC or TDBP-TAZTO) is a new brominated flame retardant (BFR) used as a replacement of classic BFR, such as tetrabromobisphenol A. TBC is supposed to be safer than classic BFRs, but reports show that it may induce a similar toxic effect. Therefore, the aim of the present study was to determine the impact of TBC on the inflammation and activation of the apoptosis process in mouse cortical astrocytes *in vitro*. Our results have shown that TBC increases caspase-1 and caspase-3 activity in mouse astrocytes *in vitro*, which suggests inflammation-induced apoptosis. Further analyses have revealed that TBC indeed increases the level of inflammation markers, *e.g.* Cat, IL-1β and IL-1βR1 proteins, but decreases the level of proliferation marker protein Ki67. However, our study has demonstrated that TBC does not change the morphology of astrocytes and does not increase the number of apoptotic bodies - a well-established marker of late apoptosis. Moreover, the concentration of 50 µM TBC also increases caspase-3 activity with no formation of apoptotic bodies. However, since 10 and 50 µM TBC have never been detected in living organisms, we can assume that the compound is safe at the low concentrations that are detected.

## INTRODUCTION

Brominated flame retardants (BFRs) have been widely used to prevent ignition or to reduce fire-related property damage to plastics, textiles, synthetic fibers, and other materials [review in [1, 2]]. Currently, the classical BFRs are replaced by an increasing number of alternative substances, such as tris-(2,3-dibromopropyl)isocyanurate (TBC or TDBP-TAZTO; CAS No. 52434-90-9) [3]. TBC is characterized by high stability and is usually used in polyolefin, polyphenyl alkene, unsaturated polyester, synthetic rubber, and fibers [4]. The annual production volume of TBC in China in the 1990s exceeded 500 metric tons [5]. Following the cessation of commercial production of some BFRs worldwide, an increasing production and use of TBC was expected in Europe, the United States of America, and Japan [3]. The high octanol water partition coefficient (Kow) of TBC indicates that it can easily accumulate both in sediments and organism tissues [reviewed in [6]]. To date, TBC has been found in river waters (2.33−163 ng/L), surface sediments (85.0 ng/g−6.03 μg/g dry weight (dw), and soils (19.6−672 ng/g dw) [3, 7]. Moreover, its concentration in the environment has been continuously increasing over the years [4, 8]. To date, it has also been shown that TBC can be accumulated in such organisms as earthworms (9.75−78.8 ng/g dw) or *Cyprinus carpio* (carp) brain tissue (12.0−646 ng/g dw) [7]. Current studies have shown that carp and mouse brains contained high levels of TBC, which indicates accumulation of this compound in the nervous system [7, 9]. According to literature data, µM concentrations of TBC has never been detected in living organisms to date. However, given the properties of TBC, such as the high octanol–air partition coefficient (Koa), high Kow, and high bioconcentration factor (BCF), an ability of this compound to bioaccumulate in living organisms may be predicted [6]. Moreover, since there are no literature data on the mechanism of the metabolism and removal of TBC from living organisms, it cannot be excluded that µM concentrations of this compound can be reached in human tissues.

TBC also induces severe hippocampal neurotoxicity, which provokes cognitive impairment and depression-like behaviors in mouse and rat models [9, 10]. Moreover, in developing primary cultures of rat cerebellum granule neurons, TBC was cytotoxic in a concentration range of 5–10 μM [11]. Similarly, TBC exerted a negative effect in undifferentiated neuroblastoma cells (SH-SY5Y) in the range of 12.5–100 µM [9]. Depending on the tissue, model, and concentration, TBC can work in different ways. Unfortunately, the mechanism of TBC action remains unclear and is still very little studied to date. However, current studies suggest that the overproduction of reactive oxygen species (ROS) is involved, followed by disturbance in antioxidant enzyme homeostasis and development of the inflammation process, which finally results in increased apoptosis, are engaged into the mechanism of action of TBC [9, 10, 12]. Unfortunately, only two studies have described that TBC may increase the level of inflammatory markers, such as tumor necrosis factor alpha (TNF-α), interleukin-6 (IL-6), and IL-1β, in rat brains and inflammatory cell infiltration and congested alveoli in BALB/c mice [10, 12].

Therefore, due to the limited knowledge of the mechanism of TBC action, in the first stage of our investigations, we decided to study the ability of this compound to activate caspase-1 and -3 after exposure of astrocytes to TBC concentrations in the range between 1 nM to 100 µM. In the second part of our study, we determined the pro-inducive inflammation properties of TBC as a model of potential acute exposure in mouse cortical astrocytes *in vitro*.

## MATERIALS AND METHODS

### Reagents

DMEM/F12 phenol red-free, trypsin, charcoal/dextran-treated fetal bovine serum (FBS), penicillin, streptomycin, glycerol, 3-[(3-cholamidopropyl)dimethylammonio]-1-propanesulfonate (CHAPS), 4-(2-hydroxyethyl)piperazine-1-ethanesulfonic acid (HEPES), ethylenediaminetetraacetic acid (EDTA), DL-dithiothreitol (DTT), TBC (269999), Hoechst 33342, calcein AM, 2’,7’ –dichlorofluorescin diacetate (H_2_DCFDA), and dimethyl sulfoxide (DMSO) were purchased from Sigma–Aldrich (St. Louis, MO, USA). The substrate for caspase-1 and caspase-3 were purchased from Merck (Darmstadt, Germany). The cytotoxicity detection kit was purchased from Roche Applied Science (Mannheim, Germany). ELISA kits for Sod1 (M2398), IL-1β (M0037), IL-1βR1 (M0017), and Cat (M2605) were purchased from Elabscience Biotechnology (Wuhan, China). ELISA kit for Ki67 (EM1473) was purchased from Fine Biotech (Wuhan, China). Stock solutions of these compounds were prepared in DMSO and added to DMEM/F12 medium. The final concentration of DMSO in the culture medium was always 0.1%.

### Primary Astrocyte Cell Culture

All procedures were performed in accordance with the National Institutes of Health Guidelines for the Care and Use of Laboratory Animals and were approved by the Bioethics Commission (Approval number: No.46/2014) as compliant with Polish law. The detailed procedure of primary astrocyte isolation has been described previously [13]. Primary astrocytes were cultured in phenol red-free DMEM/F12 medium supplemented with 10% charcoal/dextran-treated fetal bovine serum (FBS) (containing a reduced level of steroid hormones and growth factors), 50 U/mL of penicillin, and 0.05 mg/mL of streptomycin. In the experiments, the astrocytes were seeded at a density of 5 × 10^3^ per well in a 96-well plate for colorimetric and fluorometric analysis and 60 × 10^5^ per well in a six-well plate for protein analysis and cell staining. The culture medium was changed before the experiment with the TBC treatment. Then, primary neocortical astrocyte cultures were exposed to the experimental concentrations of TBC for 6, 24, or 48 h.

### Measurement of Caspase-1 and Caspase-3 Activity

The determination of the activity of caspases was chosen to assess the ability of TBC to induce inflammation (caspase-1) and apoptosis (caspase-3). The analysis was performed with the method proposed by Nicholson *et al*. [14]. After the experiments, the cell culture medium was removed and the cells were frozen in a freezer at -80 °C. After thawing, the astrocytes were lysed using lysis buffer (50 mM HEPES, pH 7.4, 100 mM NaCl, 0.1% CHAPS, 1 mM EDTA, 10% glycerol, 10 mM DTT) for 10 min at 4 °C. The lysates were incubated with the specific substrate for caspase-1 (Ac-YVAD-pNA) or caspase-3 (Ac-DEVD-pNA) at 37 °C. After 30 min, the absorbance of the lysates was measured at 405 nm in a microplate reader (FilterMax F5). The amount of the colorimetric product formed was monitored continuously for 120 min.

### Cell Staining

Hoechst 33342 and Calcein-AM staining was performed to assess changes in the morphology of the astrocytes as well as intracellular esterase activity in the mouse astrocytes 24 h after the initial treatment. The cells were seeded on a ⌀ 35 mm culture dish at the density of 1 × 10^5^ cells/dish, followed by 24-h incubation. Afterwards, the cells were treated with 100 nM, 1, 10, and 50 µM of TBC for the next 24 h. After this time, the medium was removed and the cells were washed once with warm PBS. Subsequently, the astrocytes were incubated with 10 μM Hoechst 33,342 and 4 μM calcein AM in PBS at 37 °C in an atmosphere of 5%/CO2/95% for 5 min. A fluorescence microscope (LSM 700, ZEISS) was used to visualize the stained cells.

### Measurement of ROS Production

ROS is a well-described marker of the intracellular oxidative stress occurring after treatment with certain xenobiotics. The ability of TBC to induce ROS production was determined. The method was used as in Skóra *et al*. [15]. Briefly, the cells were seeded at the density of 5 × 10^3^ per well in a 96-well plate 24 h before the experiment. After this time, the medium was removed and replaced with serum-free medium containing 5 μM of H_2_DCFDA for 30 min. Next, the medium was removed and replaced with medium containing increasing concentrations of TBC (1 nM – 100 µM). The measurement was performed after 6 h and 14 h of treatment of the astrocytes with the tested concentrations of TBC in a microplate reader at maximum excitation and emission wavelengths of 485 nm and 535 nm, respectively (FilterMax F5).

### Enzyme-linked Immunosorbent Assays (ELISA) for Ki67, Sod1, Cat, IL-1β, and IL-1βR1

The ELISA assay is based on the use of the specific antibodies against tested antigens, which facilitates determination of their amount intracellularly or in the medium with high accuracy. The ELISA method was used in this study to determine the ability of TBC to affect oxidative stress (Sod1, Cat)-, proliferation (Ki67)- and inflammation (IL-1β, IL-1βR1)-related proteins. The levels of Sod1, Cat, and IL-1βR1 in cortical astrocytes and IL-1β in the cell culture medium were determined 24 and 48 h after the treatment with TBC. Specific detection of Ki67, Sod1, Cat, IL-1β, and IL-1βR1 was carried out using enzyme-linked immunosorbent assays (ELISAs) in accordance with the protocol specified in the producer’s manual (Elabscience Biotechnology). Briefly, 96-well plates were coated with specific antibodies for mouse Ki67, Sod1, Cat, IL-1β, and IL-1βR1. Equal amounts of appropriate standards and tested samples were added for 90 min. at 37 °C. Subsequently, the wells were washed three times and biotinylated detection antibodies were applied for 60 min. Next, the washing was performed and horseradish peroxidase-conjugated avidin was added to the wells for 30 min, followed by stop solution. Afterwards, the absorbance was measured at 450 nm with a microplate reader and was proportional to the amount of Ki67, Sod1, Cat, IL-1β, or IL-1βR1 (FilterMax F5). The results were standardized to the total amount of the protein determined with the Bradford method.

### Statistical Analysis

The data are presented as the mean ± SD of four independent experiments (n = 4). The data were analyzed by one-way analysis of variance (ANOVA) followed by Tukey’s multiple comparison procedure. In the measurement of ROS and caspases, the data were analyzed using GraphPad Prism 8.0 Multi-Mode Analysis software and normalized to fluorescence (for ROS) or absorbance (for caspases and ELISAs) in a vehicle-treated control (% of control). Differences between the control and experimental groups were marked as follows: ∗p < 0.05, ∗∗p < 0.01, ∗∗∗ p < 0.001 versus control cells.

## RESULTS

### Caspase-1 and Caspase-3 Activity

**Fig. 1:**
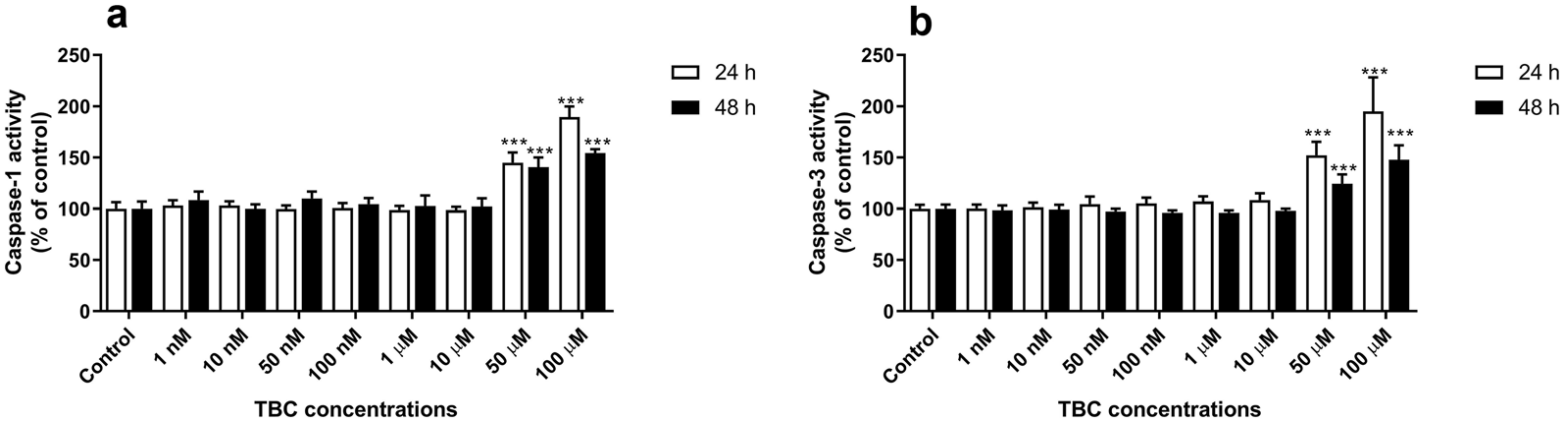
Caspase-1 (**a**) and caspase-3 (**b**) activity after the 24- and 48-h exposure of mouse primary astrocytes *in vitro* to 1, 10, 50, 100 nM and 1, 10, 50 100 µM TBC. Data are expressed as means ± SD of four independent experiments (n = 4). **p < 0.01 and ***p < 0.001 vs. the control cells.

Our experiments showed that, after the 24- and 48-h exposure of the astrocytes to 1, 10, 50, 100 nM and 1, 10, 50, 100 µM TBC, only the highest micromolar concentrations increased caspase-1 activity. Especially, the 24-h exposure to 50 and 100 µM produced a 44.83 and 89.30% increase in caspase-1 activity, respectively, compared to the control (Fig. 1a). Similarly, the 48-h treatment with 50 and 100 µM increased caspase-1 activity by 40.36 and 54.29%, respectively, compared to the control (Fig. 1a).

Our experiments showed that only the highest concentrations of TBC increased caspase-3 activity after the 24- and 48- of exposure of the astrocytes to the increasing concentrations of the compound. Especially, the 24-h exposure to 50 and 100 µM increased caspase-3 activity by 52.03 and 94.88%, respectively, compared to the control. However, in the range of 100 nM to 10 µM TBC, the activity of caspase-3 increased slightly but not statistically significantly (Fig. 1b). After the 48-h exposure to 50 and 100 µM, caspase-3 increased by 24.39 and 47.72%, respectively, compared to the control (Fig. 1b).

### Hoechst 33342 and Calcein-AM Staining

**Fig. 2:**
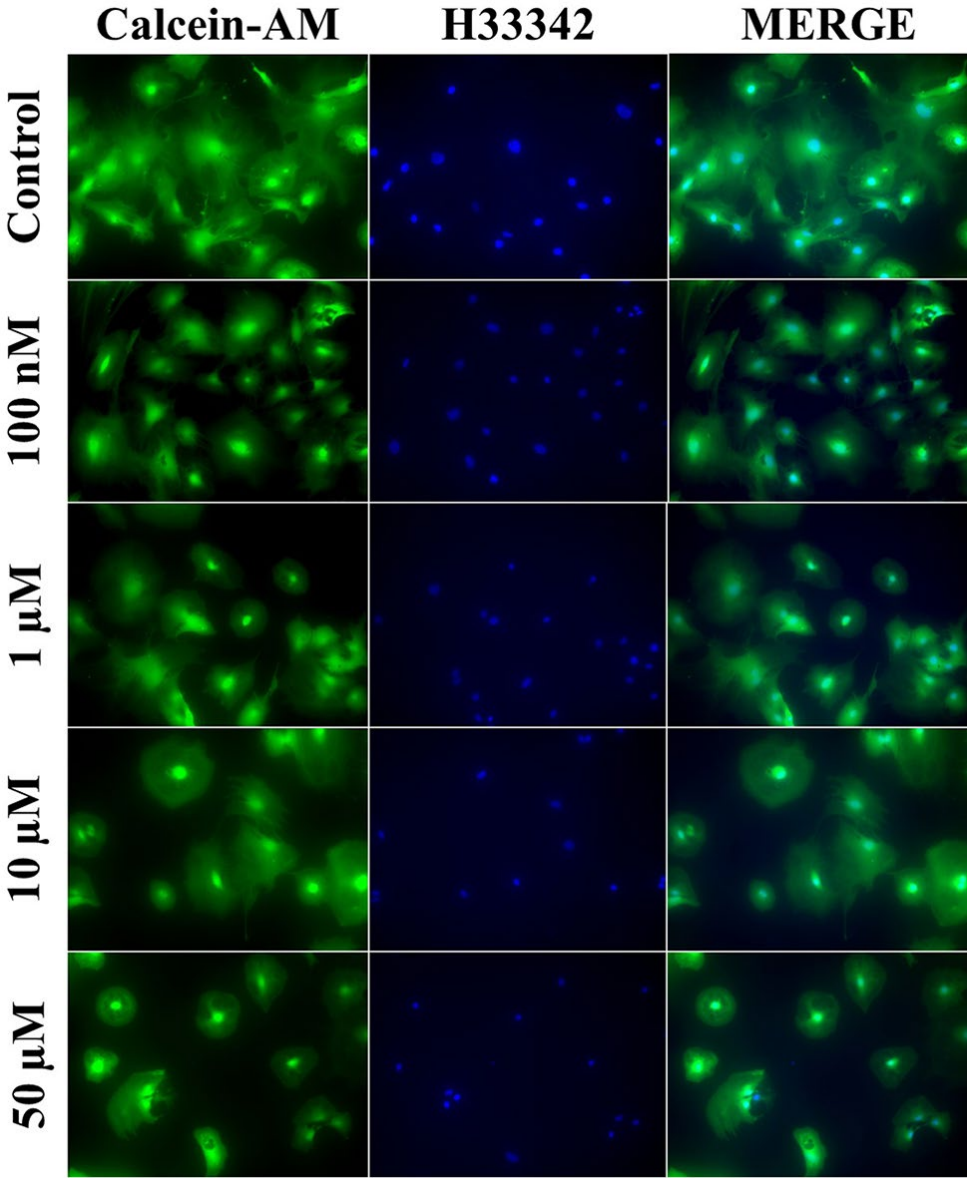
Cell staining with Hoechst 33342 and Calcein-AM after the 24-h exposure to 100 nM, 1, 10, and 50 µM TBC. The images were captured at 200× magnification.

Our experiments showed that, after the 24-h exposure to TBC in the concentration range from 100 nM to 50 µM, the studied compound did not cause changes in the morphology of the astrocytes visualized with the use of the Hoechst 33,342 dye (Fig. 2). The calcein AM staining revealed no changes in the cell metabolism after the 24-h TBC treatment in the concentration range from 100 nM to 50 µM. However, we observed that, at the concentrations of 10 and 50 µM, the number of cells decreased, compared to the control (Fig. 2).

### ROS Production Measurement

**Fig. 3:**
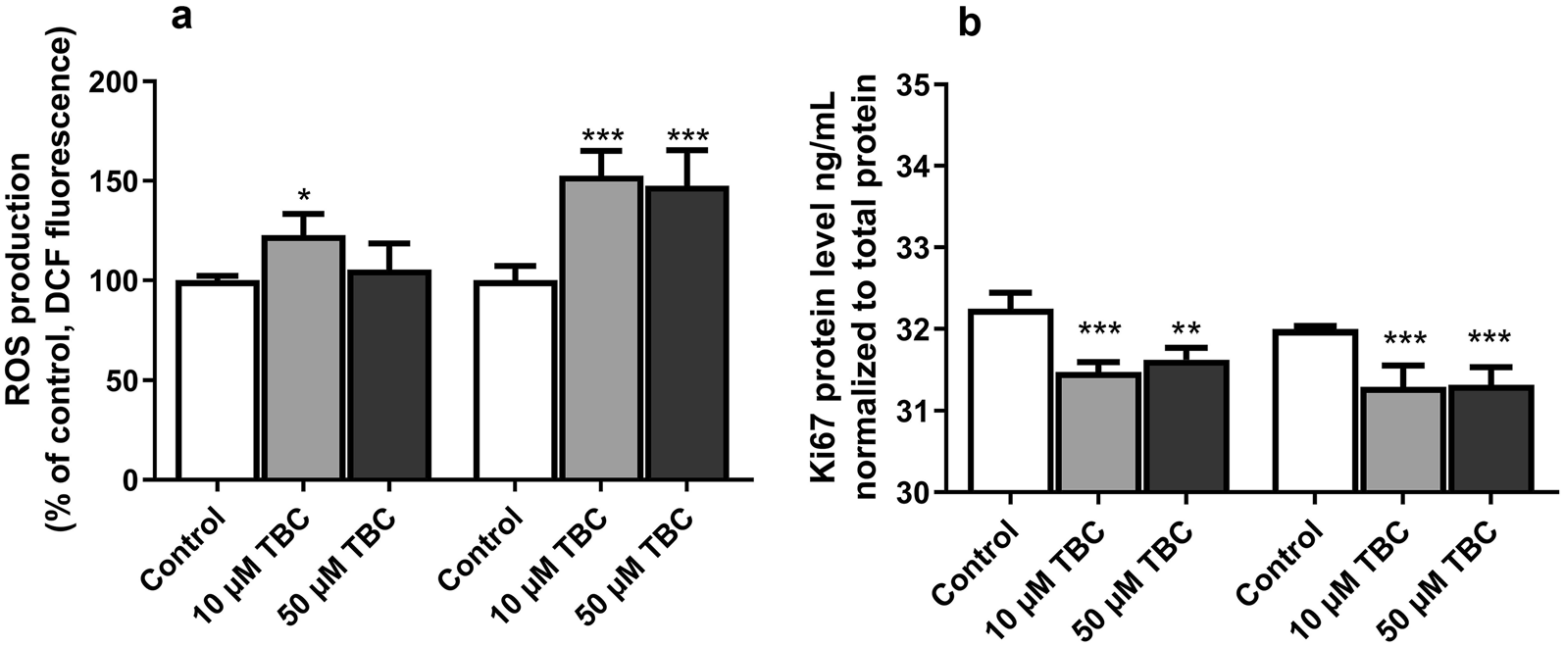
Effect of 10 and 50 µM TBC on the ROS (**a**) production after the 6- and 24-h exposure of primary mouse astrocytes *in vitro*. Effect of 10 and 50 µM TBC on the level of Ki67 (**b**) protein expression after the 24- and 48-h exposure. The Ki67 protein was measured with the ELISA method. Data are expressed as means ± SD of four independent experiments (n = 4). **p < 0.01 and ***p < 0.001 vs. the control cells.

After the 6-h exposure of the astrocytes to 10 and 50 µM TBC, only 10 µM TBC increased ROS production by 22.67%, compared to the control. Similarly, after the 24-h exposure to 10 and 50 µM, TBC increased ROS production by 52.58 and 47.53%, respectively, compared to the control (Fig. 3a).

### ELISA Protein Measurements

After the 24-h exposure of the mouse astrocytes to 10 and 50 µM TBC, both concentrations decreased the level of the Ki67 protein by 0.77 and 0.62 ng/mL, respectively, compared to the control. Similarly, after the 48-h treatment of the mouse astrocytes with 10 and 50 µM TBC, both concentrations decreased the level of the Ki67 protein by 0.70 and 0.67 ng/mL, respectively, compared to the control (Fig. 3b).

**Fig. 4:**
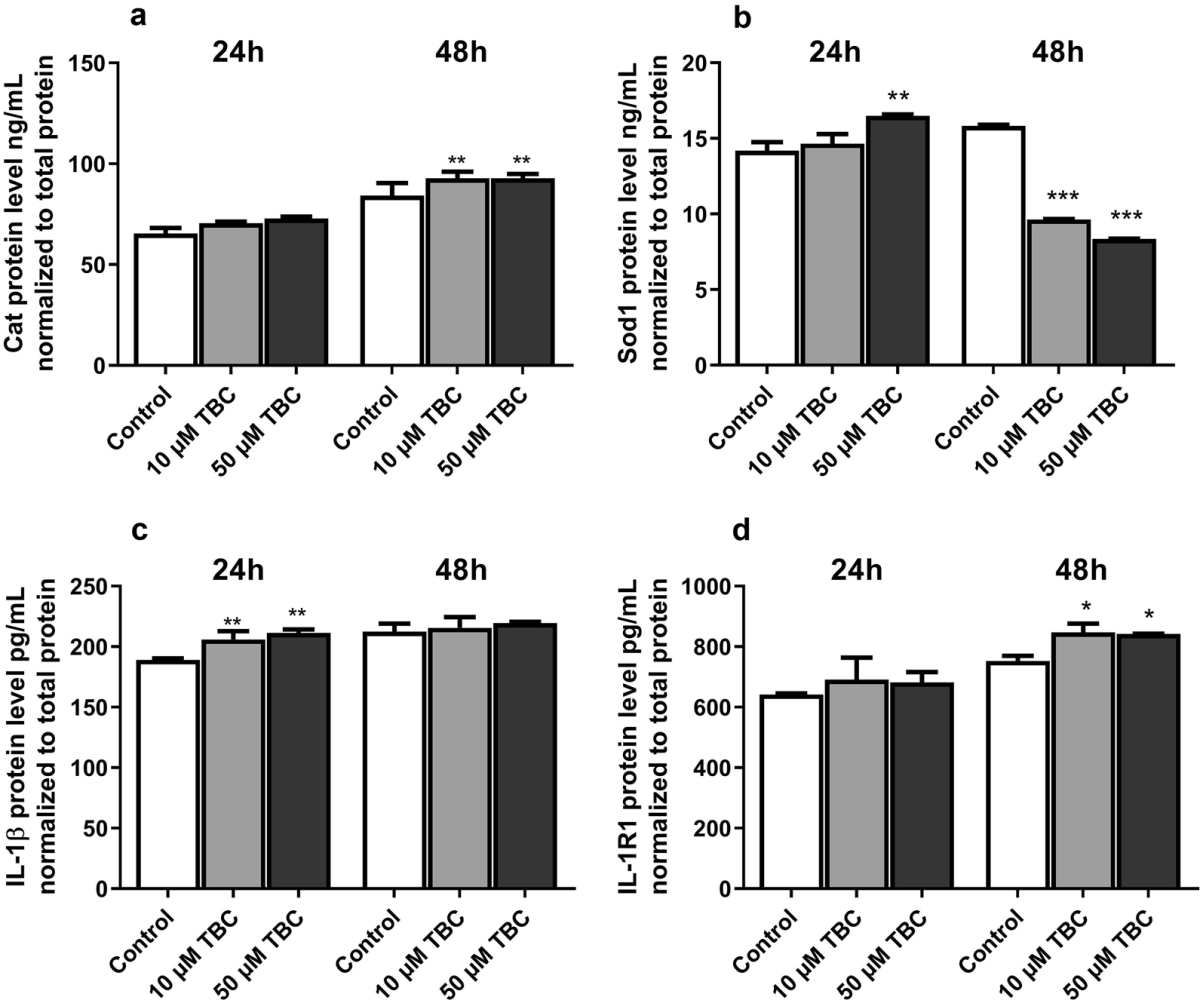
Effect of 10 and 50 µM TBC on the level of Cat (**a**), Sod1 (**b**), IL-1β (**c**), and IL-1βR1 (**d**) protein expression after the 24- and 48-h exposure. The Cat, Sod1, IL-1β and IL-1βR1 proteins were measured with the ELISA method. Data are expressed as means ± SD of four three independent experiments (n = 4). **p < 0.01 and ***p < 0.001 vs. the control cells.

The experiments showed that the 24-h exposure to both TBC concentrations (10 and 50 µM) increased the Cat protein level slightly but not statistically significantly. However, after 48 h, TBC increased the Cat level by 8.61 (10 µM TBC) and by 8.70 (50 µM TBC) ng/mL, compared to the control (Fig. 4a).

After the 24-h exposure, only 50 µM TBC increased the Sod1 protein expression by 2.30 ng/mL, compared to the control. However, after the 48-h treatment, both studied concentrations (10 and 50 µM TBC) decreased the level of Sod1 by 6.17 and 7.45 ng/mL, respectively, compared to the control (Fig. 4b).

Our experiments showed that the 24-h exposure of the astrocytes to 10 and 50 µM increased the IL-1β level in the culture medium (an increase by 17.06 and 22.34 pg/mL, respectively, compared to the control). However, no statistically significant changes were observed after 48 h (Fig. 4c).

After the 24-h exposure to TBC, no statistical significant changes in the level of IL-1βR1 in the mouse astrocytes *in vitro* were observed. Our experiments showed that the TBC concentrations of 10 and 50 µM caused a statistically significant increase in IL-1βR1 only after the 48-h exposure (an increase by 93.92 and 89.06 pg/mL, respectively, compared to the control) (Fig. 4d).

## DISCUSSION

It is worth noting that µM concentrations have never been detected in living organisms. However, Jarema *et al*. showed that acute exposure (10 µM) of *Danio rerio* larvae with five known BFRs and bisphenol derivatives – triphenyl phosphate (TPHP), tetrabromobisphenol A (TBBPA), 2-ethylhexyl diphenyl phosphate (EHDP), isopropylated phenyl phosphate (IPP), and t-butylphenyl diphenyl phosphate (BPDP) caused changes in the behavior of this organism [16]. Therefore, due to the possible bioaccumulation of TBC and given the above-mentioned study results, we decided to determine the possible consequences of severe TBC poisoning at µM concentrations. Our experiments showed that TBC increased the caspase-3 activity only at the highest µM concentrations (50 and 100 µM). It is worth noting that µM concentrations have never been detected in living organisms. However, due to the possible bioaccumulation of TBC, we decided to study the possible consequences of severe TBC poisoning at µM concentrations. However, the Hoechst 33342 dye and calcein AM cell staining revealed no changes in the cell morphology with a slight decrease in the cell number. Moreover, our analysis of the level of the Ki67 protein, which is a well-established marker of proliferation, showed that 10 and 50 µM of TBC decreased its expression in both time intervals. In the same concentration range, we observed a significant increase in the amount of ROS. As reported in previous studies, TBC can act in a dose- and cell-type specific manner with a toxic effect at micromolar concentrations. However, in cells derived from the nervous system, the TBC toxicity depended on the degree of cell differentiation. In the neuroblastoma (SH-SY5Y) cell line, TBC was toxic in a broad range of concentrations 1 nM to 100 µM in undifferentiated and differentiated cells and up to day 14 [9, 17]. In turn, Qu *et al*. reported TBC toxicity in primary cultures of rat neurons developing from cerebellar granule cells in the concentration range of 5–10 µM [11]. Interestingly, no TBC toxicity was detected in cells with high expression of antioxidant enzymes such as human hepatocarcinoma (Hep-G2) cells [7].

To date, Ye *et al*. have described an increase in the caspase-3 expression after TBC treatment in the hippocampus of adult rats [10]. Moreover, the authors correlated the observed apoptosis with an increase in the ROS production [10]. Other authors, *e.g.* Dong *et al*. described an increase in the expression of the Bax protein and reduced expression of Bcl-2 in SH-SY5Y cells after stimulation with 12.5–50 µM concentrations of TBC, which was accompanied by an increase in the production of malondialdehyde (MDA) and superoxide dismutase (SOD) activity and a decrease in the amount of glutathione (GSH) [9]. Similarly, Li *et al*. described an increase in the level of apoptosis measured by an increase in the mRNA expression of *p53* and *Bcl-2* genes in livers of BALB/c mice treated with TBC [12]. Szychowski *et al*. also reported an increase in caspase-3 activity and ROS production caused by TBC in a wide range of concentrations (1 nM to 100 µM) in sensitive SH-SY5Y cells differentiated for 14 days [17]. Qi *et al*. described that TBC acts in the Hep-G2 cell line via the mitochondrial pathway and involvement of death receptors [18]. This indicates that TBC induces apoptosis in a ROS-dependent manner with engagement of the p53 protein. However, based on the above-cited research, it can be concluded that TBC is also able to engage in the destabilization of the mitochondrial redox balance, leading to apoptosis. However, as reviewed by Mittal *et al*., ROS is one of the main inflammation-inducing factor [19].

To prove the suspected involvement of ROS and antioxidant enzymes in the TBC mechanism of action and initiation of inflammation, in the last part of our study, we decided to measure the level of Sod1, Cat, IL-1β, and IL-1βR1 proteins and caspase-1 activity. It is well described that caspase-1 cleaves pro-IL-1β to active IL-1β [20]. Moreover, our study is the first to describe the increase in caspase-1 activity after the TBC treatment. In our experimental model, an increase in the IL-1β level was observed after the application of 10 and 50 µM TBC. In turn, an increase in the activity of caspase-1 was observed only at the 50 and 100 µM TBC concentrations. We believe that the results obtained using the ELISA method are more accurate due to the presence of the specific antibodies against IL-1β, while the absence of changes in the caspase-1 activity assay in the astrocytes treated with 10 µM of TBC may be an effect of the lower sensitivity of this method. Due to the slight discrepancy between the lack of caspase-1 activity and the increase in IL-1β in the group treated with 10 µM TBC, we cannot exclude that it is activated in a different non-classical way at this concentration. To date, it has been shown that pro-IL-1β can be activated to IL-1β without caspase-1 involvement, *e.g.* by caspase-8, elastase, chymase, and proteinase 3 [21]. Moreover, Guma *et al*. proved the activation of functional IL-1β in caspase 1 knock-out (Casp1−/−) mice, which supports the above-stated hypothesis and may partially explain the compensation effect in this aspect by other enzymes [22]. To date, Ye *et al*. have described that TBC induces an increase in tumor necrosis factor alpha (TNF-α), IL-6, and IL-1β (pro-inflammatory cytokines) protein levels in the rat hippocampus after treatment with TBC in a dose of 5 and 50 mg/kg [10]. Moreover, Li *et al*. have shown that TBC induces dose-dependent hyperplasia of pulmonary alveolar epithelium, bronchial congestion, infiltration of inflammatory cells, and mitochondrial swelling in the lungs of BALB/c mice [12]. The present experiments demonstrated that TBC increased the Cat and IL-1βR1 protein expression in the cells and the level of IL-1β in the culture medium in both studied time intervals. However, after the 24-h exposure, we observed a slight increase in the Sod1 protein expression, while a decrease in its level was observed after 48 h. Unfortunately, there are only few studies concerning the impact of TBC on antioxidant enzymes or inflammation markers. Ye *et al*. correlated apoptosis in the rat hippocampus with an increase in the MDA level (a well-described marker of oxidative damage to fatty acids) and a decrease in GSH and SOD activities [10]. Similarly, Dong *et al*. described increased apoptosis markers in SH-SY5Y cells after stimulation with TBC at concentrations ranging from 12.5 to 50 µM, which was accompanied by an increase in the production of MDA and SOD activity; additionally, Dong *et al*. described a decrease in the amount of GSH [9]. Finally, in our experimental model, we did not notice dose-dependent changes in the studied parameters between the 10 and 50 µM variants. The observed phenomenon may be related to a different concentration-dependent mechanism of action of TBC. Moreover, as shown by the fluorescent microscopy-based analysis (calcein AM staining), the higher concentration of TBC (50 µM) reduces the number of cells, which may result in a smaller amount of studied proteins and lack of dose dependency. Moreover, Espugas *et al*. have recently shown a similar tendency in neuroblastoma cells (SH-SY5Y) treated with some novel BFRs, namely no dose-dependent changes in the cell viability in the concentration range between 2.5 µM and 20 µM, followed by only slightly changes in certain mRNA expression [23]. Therefore, it may be supposed that TBC acts in a time-, concentration-, tissue-, and cell-dependent manner. Therefore, we can assume that our data are mainly consistent with the current state of knowledge of the TBC mechanism of action.

## CONCLUSIONS

Our results have demonstrated that TBC increases caspase-1 and caspase-3 activity in mice, which suggests inflammation-induced apoptosis. The further analyses revealed that TBC indeed increased the level of such inflammation markers as Cat, IL-1β and IL-1βR1 proteins and decreased the level of the proliferation marker (Ki67). Additionally, our study revealed that TBC did not change the morphology of astrocytes and did not increase the number of apoptotic bodies (a well-known marker of late apoptosis). It was found that 10 and 50 µM TBC induced an increase in the inflammatory markers, but 50 µM TBC also increased caspase-3 activity with no apoptotic bodies. However, since the astrocyte response is different at 10 and 50 µM, we can assume that the mechanism of action may be different at these two concentrations. Given the increasing levels of TBC in the environment, this may pose a serious health problem.

## Data Availability

Original data available for request.

## Abbreviations

BFRs: Brominated flame retardants
CHAPS: 3-[(3-Cholamidopropyl)dimethylammonio]-1-propanesulfonate
DMSO: Dimethyl sulfoxide
DTT: Dithiothreitol
EDTA: Ethylenediaminetetraacetic acid
ELISA: Enzyme-linked immunosorbent assays
FBS: Fetal bovine serum
GSH: Glutathione
H_2_DCFDA: 2,7′-Dichlorodihydrofluorescein diacetate
HEPES: 4-(2-Hydroxyethyl)piperazine-1-ethanesulfonic acid
IL-6: Interleukin-6
LDH: Lactate dehydrogenase
MDA: Malondialdehyde
PBS: Phosphate buffered saline
ROS: Reactive oxygen species
SOD: Superoxide dismutase
TBC: Tris-(2,3-dibromopropyl)isocyanurate
TNF-α: Tumor necrosis factor alpha
